# Novel Microglia-based Therapeutic Approaches to Neurodegenerative Disorders

**DOI:** 10.1007/s12264-022-01013-6

**Published:** 2023-01-03

**Authors:** Lijuan Zhang, Yafei Wang, Taohui Liu, Ying Mao, Bo Peng

**Affiliations:** 1 Department of Neurosurgery, Huashan Hospital, Institute for Translational Brain Research, State Key Laboratory of Medical Neurobiology, MOE Frontiers Center for Brain Science, Fudan University, Shanghai, 200040 China; 2 Co-innovation Center of Neuroregeneration, Nantong University, Nantong, 226001 China

**Keywords:** Microglia, Neuroinflammation, Multifunction, Microglial replacement, Neurodegeneration

## Abstract

As prominent immune cells in the central nervous system, microglia constantly monitor the environment and provide neuronal protection, which are important functions for maintaining brain homeostasis. In the diseased brain, microglia are crucial mediators of neuroinflammation that regulates a broad spectrum of cellular responses. In this review, we summarize current knowledge on the multifunctional contributions of microglia to homeostasis and their involvement in neurodegeneration. We further provide a comprehensive overview of therapeutic interventions targeting microglia in neurodegenerative diseases. Notably, we propose microglial depletion and subsequent repopulation as promising replacement therapy. Although microglial replacement therapy is still in its infancy, it will likely be a trend in the development of treatments for neurodegenerative diseases due to its versatility and selectivity.

## Introduction

Microglia were first described by the Spanish neuroscientist Pío del Río-Hortega in 1919 [1] and were gradually found to be maverick immune cells in the central nervous system (CNS) that are both specialized and diverse. In mice, microglia originate from a pool of primitive macrophages in the yolk sac on embryonic (E) day 8.5 [2]. During development, microglia survive through colony-stimulating factor 1 receptor (CSF1R) signaling [3], and further differentiation depends on the activity of the transcription factors *PU.1* and interferon regulatory factor 8 [4]. In the adult brain, microglia have a ramified morphology and express *TMEM119, CD11b,* and *P2ry12/P2ry13*, which participate in the immune response [5]. In the normal brain, microglia are highly dynamic and perpetually scan the CNS [6], influencing fundamental processes such as protein aggregation, soluble factor secretion, phagocytosis, and neural circuit refinement [7]. In the context of CNS injury, microglia undergo complex, multistage activation and respond through signaling molecules such as cytokines and chemokines for subsequent tissue repair [8].

The recent introduction of single-cell RNA sequencing and single-nucleus RNA sequencing analyses has greatly advanced our knowledge of microglial responses in neurodegenerative diseases [9–11], leading to the identification of special microglial subsets associated with neurodegeneration in both mouse models and human specimens. For example, using transcriptional single-cell sorting, Keren-Shaul *et al*. identified disease-associated microglia (DAMs) in proximity to amyloid-β (Aβ) plaques involved in the immune response in AD mouse brains [12]. Krasemann *et al.* reported that the microglial neurodegenerative phenotype is characterized by genetic upregulation of ApoE signaling and suppression of TGFβ signaling, and this phenotype partially overlaps with that of the DAMs found in AD mouse models [13]. Another study reported that activated response microglia are composed of specialized subgroups overexpressing MHC (major histocompatibility complex) type II genes and show strongly upregulated expression of AD risk genes in the AD mouse model [14]. By using single-cell RNA sequencing, Mathys *et al.* identified multiple disease stage-specific cell states, including IFN-I and IFN-II co-regulated DAMs in a mouse model of severe neurodegeneration with AD-like phenotypes [15]. Microglial features determined from snRNA-seq data from brain specimens of humans with neurodegenerative diseases show considerable heterogeneity and appear to be different from those in mouse models. For example, Friedman *et al*. analyzed RNA profiles in the brains of AD patients and showed that they were not replicated in animal models or individuals with early-onset and late-onset AD [16]. They also identified multiple dimensions of CNS myeloid cell activation that occur differentially in response to specific disease conditions. Beyond a detailed description of different microglial subsets, it will be essential to investigate additional biological characteristics of microglia.

It has long been assumed that microglia are long-lived cells that persist throughout the entire lifespan of mice or humans under physiological conditions. Lawson *et al.* [17] used [3H] thymidine incorporation and autoradiography and reported that 0.05% of the microglia proliferated within 1 hour. Askew *et al.* [18] analyzed the proliferation of resident microglia by using BrdU incorporation and found that 0.69% of the total microglial cells proliferated after a single pulse of BrdU (the estimated value in humans is ~2%). Therefore, at the individual cell level, microglia replicate frequently. Regarding pathological conditions, Tay *et al.* [19] established a new multicolor fluorescence fate mapping system and found that CNS pathology shifts from random self-renewal of the microglial network toward a rapid expansion of selected microglial clones. Füger *et al*. [20] imaged individually-labeled microglia in APP/PS1 mice and found increased microglial turnover in the presence of amyloid lesions.

The maintenance and local expansion of microglia are solely dependent on the self-turnover of CNS-resident cells under normal conditions. When microglia are depleted by inhibiting CSF1R signaling, newborn microglia rapidly replenish the population in the whole brain in a short time after drug withdrawal [21]. Importantly, the repopulated microglia are solely derived from residual microglia [22]. In the context of disease, numerous cells of the myeloid lineage appear in the nervous parenchyma in pathological settings, such as in AD models and experimental autoimmune encephalitis (EAE) models. Myeloid cells enter the nervous parenchyma, and some of them appear to transform into cells resembling microglia [23].

In this review, we highlight new evidence demonstrating the unique and diverse properties of microglia in the healthy and diseased brain, including in the context of Alzheimer’s disease (AD), Parkinson’s disease (PD), and multiple sclerosis (MS). In addition, we elaborate on the theoretical basis of microglial replacement and other microglia-based therapies for translational clinical applications.

## The Multifaceted Functions of Microglia in the CNS

**Fig. 1:**
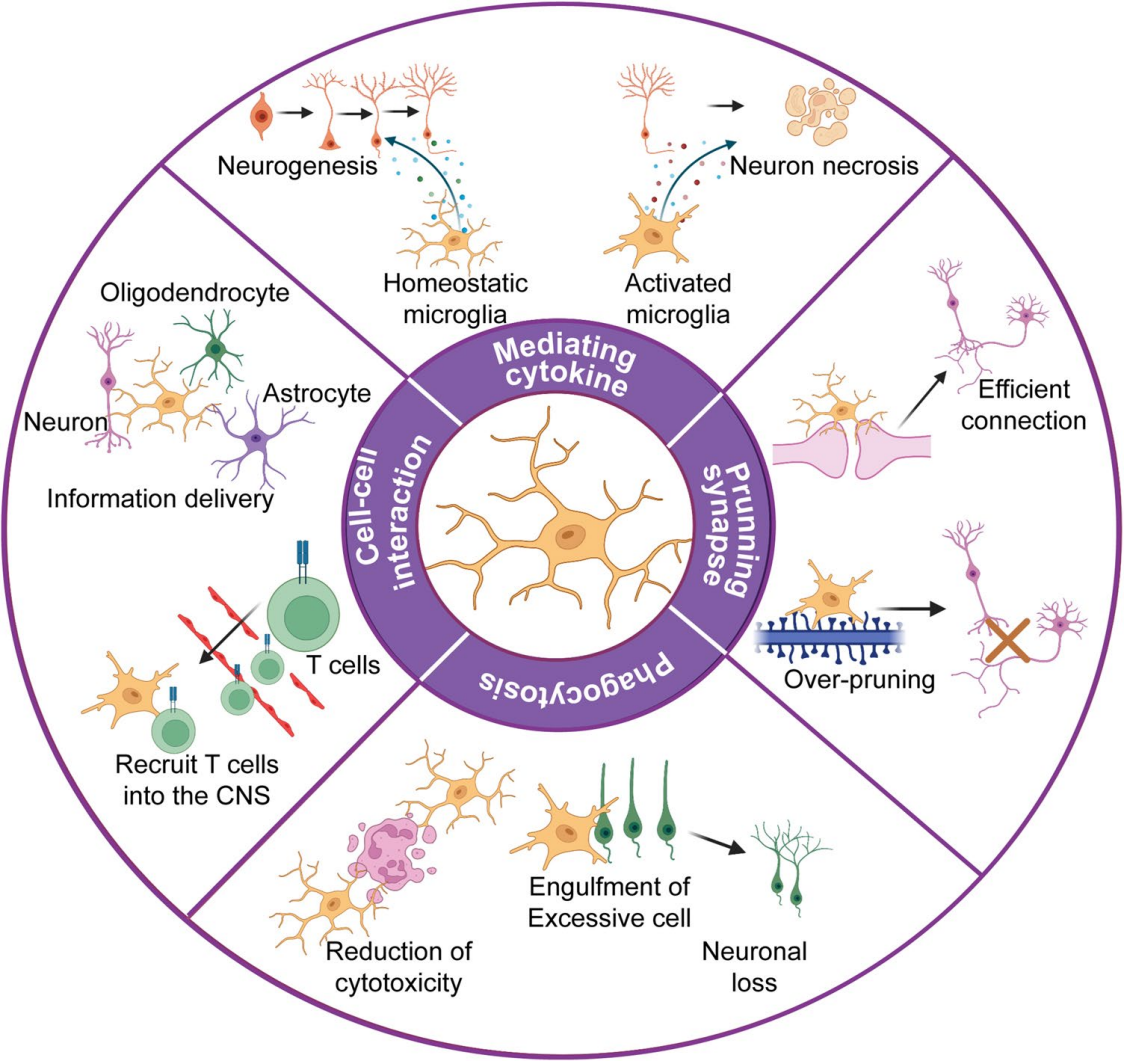
Microglia play multifaceted roles in the CNS. Upper: homeostatic microglia promote neurogenesis by releasing neurotrophic factors, whereas inflammation-associated microglia lead to neuronal cell death. Right: microglia are crucial for synaptic pruning and circuit refinement. Synaptic over-pruning induces synaptic dysfunction, which is found in neurodegenerative diseases. Lower: phagocytosis is essential for CNS homeostasis. Microglia can remove apoptotic cells and prevent the release of toxic intracellular contents. However, hyperactive microglia engulf newborn neurons, leading to a decrease in the number of mature neurons. Left: microglia–neuron/oligodendrocyte/astrocyte crosstalk promotes neurogenesis and axon formation. Microglial hyperactivation disrupts the blood-brain barrier and recruits peripheral cells into the brain during infection.

The biological functions of microglia in the CNS are multifaceted. They exert both neuroprotective and neurotoxic effects (Fig. 1). In a healthy brain, microglia are responsible for mediating inflammation, regulating a broad spectrum of cellular responses, and eliminating microbes, dead cells, and protein aggregates [24]. Microglia were originally known for their immune functions. They are the first responders to neuroinflammation and rapidly adapt their phenotypes and functions to fit the brain milieu [1]. Microglia secrete cytokines and chemokines that contribute to various aspects of immune responses in the CNS. During infection, chronic activation of microglia leads to sustained production of proinflammatory cytokines that cause damage to the surrounding neurons. This notion has been confirmed in various brain disorders, such as AD, PD, and MS. For example, in AD patients, the neurotoxic factors produced by activated microglia cause nitration of tyrosine 10 of the Aβ peptide, which promotes the aggregation of senile plaques [25]. In PD patients, inflammatory mediators such as IL-1β and IL-6, and epidermal growth factor released by activated microglia, can lead to the death of dopaminergic neurons [26]. PET studies have indicated that motor disability is correlated with the abundance of activated microglia in MS patients [27].

In addition to immune function, as resident macrophages in the brain, microglia vigilantly perform surveillance to ensure parenchymal homeostasis. Once they migrate into the brain parenchyma, microglia actively communicate with other cells. Early after birth, microglia release neurotrophic factors such as IGF-1 to promote neuronal survival and axon fiber formation [28]. Brain-derived neurotrophic factors released by activated microglia promote neurogenesis in the CNS [29]. At the same time, microglia are also well poised to induce programmed cell death, which has been demonstrated to be CD11b-dependent [30]. Afterward, microglia clean up the resulting cellular debris by phagocytosis. Microglial phagocytosis is mediated by signaling *via* triggering receptors expressed on myeloid cell-2 (TREM2) [31]. In addition, microglia participate in maintaining synaptic homeostasis *via* neuronal pruning and synaptogenesis [32, 33]. Oligodendrocytes, another important group of cells that are pivotal for myelin production around axons, are deeply involved in microglial maturation [34, 35]. In addition, microglia establish a delicate balance with astrocytes and are responsible for the maintenance of neuronal functions and brain homeostasis [36, 37].

**Fig. 2:**
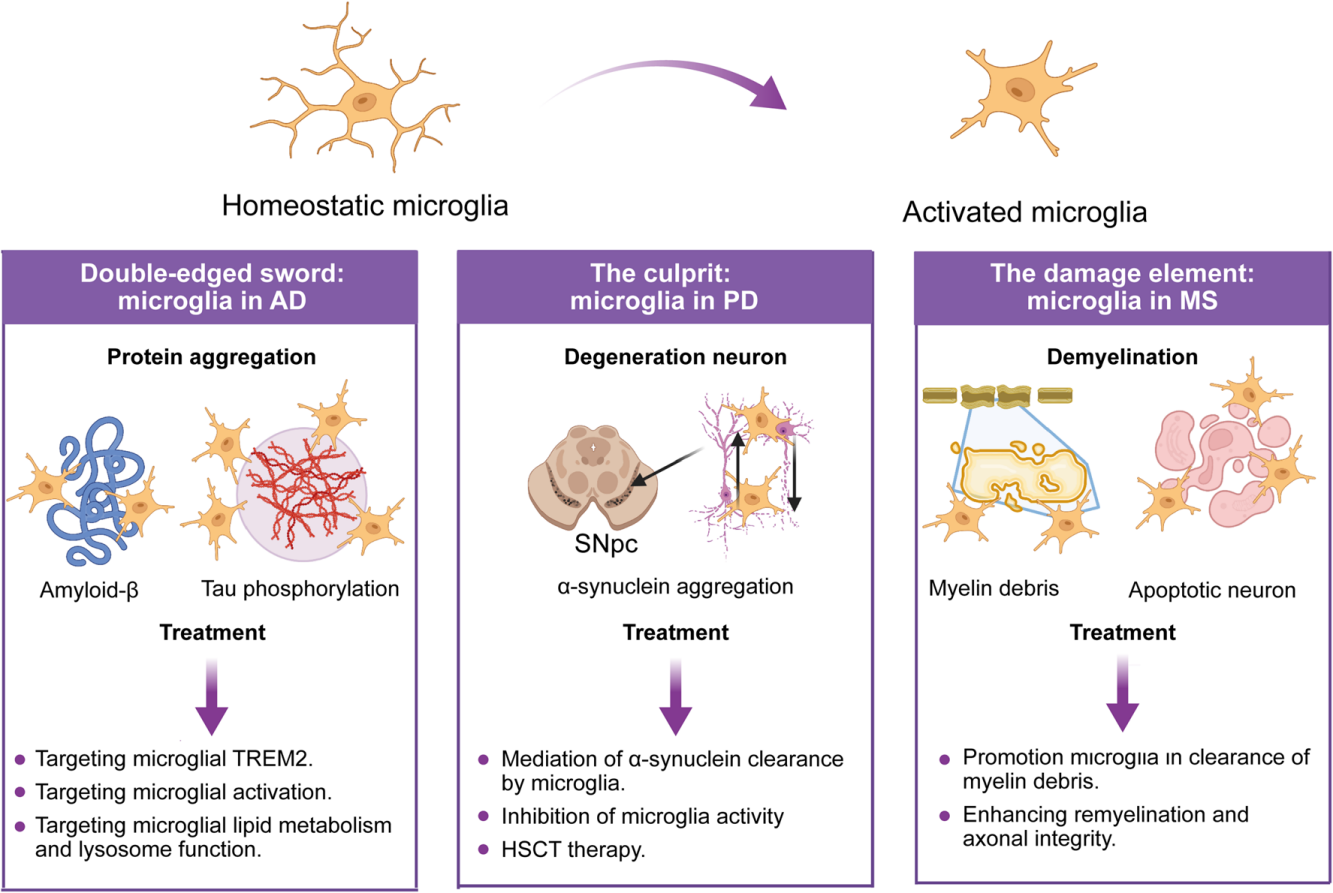
Microglia are active sensors and versatile effector cells in the brain under pathological conditions. Microglia constantly screen the microenvironment in the CNS and transform into a reactive state under certain pathological conditions. However, the tilt toward harmful or beneficial outcomes of microglial activation varies between neurological diseases. (1) Microglia in AD. AD is typified by the accumulation of extracellular Aβ peptides and intracellular deposits of hyperphosphorylated tau. Microglia are committed to an inflammatory response and can engulf Aβ deposits and impair cells. Microglial treatments for AD include targeting the microglial inflammatory response, TREM2 activation, lipid metabolism, and lysosomal function. (2) Microglia in PD. PD is primarily characterized by the death of dopaminergic neurons that project from the SNpc. Reactive microglia are involved in dopaminergic cell death as proinflammatory mediators. Reactive microglia also block the delivery of α-syn to the nigrostriatal tract. Microglia-based treatments for PD include the promotion of α-synuclein transfer and clearance, anti-inflammatory treatments, and HSCT therapy. (3) Microglia in MS. MS is a chronic inflammatory disease that leads to focal plaques of primary demyelination and diffuse neurodegeneration. Active demyelination is usually associated with microglial activation. Microglial treatments for MS include the regulation of microglial inflammatory signaling, the depletion of microglia to reduce demyelination, and the targeting of microglial phagocytosis. AD, Alzheimer's disease; PD, Parkinson’s disease; MS, multiple sclerosis; SNpc, substantia nigra pars compacta; α-syn, alpha-synuclein; HSCT, hematopoietic stem cell transplantation.

Microglia are active sensors and versatile effector cells not only in healthy brains but also in diseased brains. In the following section, we emphasize the role of microglia in neurodegenerative diseases of the CNS, including AD, PD, and MS (Fig. 2).

## Microglia are a “Double-edged Sword” in the Progression of AD

AD is the major cause of dementia and is mainly characterized by progressive neuronal loss followed by cognitive impairment. The current understanding is that AD is closely associated with genetic, aging-related, and environmental factors [38–40]. The main pathological features of AD include the deposition of insoluble Aβ peptides, as well as the aggregation of hyperphosphorylated tau protein, which lead to the formation of neurofibrillary tangles in the brain [38–40].

Reactive gliosis and the inflammatory response are hallmarks of AD. Microglia-mediated inflammation is a 'double-edged sword', performing both detrimental and beneficial functions. For example, activated microglia in AD mice show upregulation of inflammatory markers such as CD36, CD14, CD11c, MHC-II, and iNOS, which might disturb neuronal functions and lead to cognitive decline [41]. Clinical imaging studies have reported negative correlations between microglial activation measured by [11C]PK11195 PET and the structural integrity and functional activity of the brain in AD patients [42], whereas some inducers of inflammation, such as lipopolysaccharide (LPS), activate microglia to promote the degradation of Aβ [43]. Microglia interact with Aβ and amyloid-beta precursor protein (APP) through specific pattern-recognition receptors, including CD14, CD36, and Toll-like receptors, which are strongly expressed on the microglial surface [44]. This interaction increases microglial phagocytosis, resulting in the clearance of Aβ from the brain [45, 46]. As an example, the phagocytic index and total Aβ load are higher in IL-1α (+) and IL-1Ra (+) microglia and microglia producing TNF-α and IL-1β are associated with a lower Aβ load and phagocytic index in AD mice [47]. Therefore, strategies need to be developed to modulate the activation of microglia by inhibiting the release of inflammatory factors. Preclinical trials have shown that genetic and pharmacological blockade of TNF effectively alleviates amyloid pathology and tau phosphorylation [48]. Intraperitoneal administration of an antibody blocking IL-1 receptors has also been shown to decrease the activity of several tau kinases in the brains of 3×Tg-AD mice [49]. However, further clinical trials are needed to assess the safety and efficacy of this antibody in humans.

Differences exist between microglia in humans and mouse AD models. At the site of neurodegeneration, plaques are surrounded by activated microglia named DAMs [12]. Genome-wide studies have shown that some genes, such as *APOE4*, *TREM2,* and *CD33*, have unique expression patterns in DAMs [13, 50, 51]. However, no DAM signature has been found in human AD *via* single-nucleus RNA sequencing (snRNA-seq), and only a few microglial genes, including APOE and SPP1, have been identified [52]. In another study of patients, the differentially expressed microglial genes included *EEF1A1, GLULL, KIAA1217, LDLRAD3*, and *SPP1*, which differ from the characteristic genes of DAMs [53]. DAMs have been reported in other conditions, including aging, ALS, and frontotemporal dementia. The scRNA-seq profiles of dissected human brain specimens from MS patients have revealed various microglial populations expressing DAM genes [54]. Given the heterogeneity of brain pathology, certain DAM genes may be involved in different diseases. Research into the treatment of AD should consider the feasibility of implementing this approach in the clinic.

Overall, microglia, as a “double-edged sword” in the progression of AD pathology, have both beneficial and detrimental effects. A better understanding of the role of microglia in the progression of AD pathology is needed, as microglia could be a target and a tool for AD treatment.

## Microglia: The Culprit in PD

PD is the second most common neurodegenerative disorder and is characterized by motor and non-motor symptoms. The widely recognized pathology of PD involves the degeneration of dopaminergic neurons in the substantia nigra pars compacta (SNpc) and the presence of cytoplasmic protein aggregates known as Lewy bodies in the remaining dopaminergic neurons [55]. In the early stages of PD, α-synuclein undergoes aggregation and fibrillization, which can lead to neurotoxicity [56, 57]. In late-onset PD, a large genome-wide association study identified a single nucleotide polymorphism in the human leukocyte antigen (HLA-DRA) gene. HLA-DR is a genetic risk factor for PD patients and is expressed by antigen-presenting cells such as microglia [58]. Another study showed that HLA-DR-positive microglia (“reactive” microglia) are also found in the substantia nigra of PD patients [59].

Although molecular profiling of microglia in animal models of PD has yet to be attempted, activated microglia are known to be prevalent in mouse models and PD patients. There is emerging evidence that microglial dysfunction contributes to PD pathogenesis and progression [60], as exhibited by a weakened inflammatory response, reduced phagocytosis, and decreased interactions with neurons. In a PD mouse model, neuroinflammation and associated “reactive” microglia precede the onset of astrogliosis and dopaminergic cell death [61]. Many studies have described reactive microglia in postmortem brain samples from PD patients. Jyothi *et al*. reported that the microglial count showed a biphasic increase in the vicinity of the few remaining nigral dopamine neurons and that microglia displayed a morphology characteristic of activated cells in PD patients [62]. A PET study using [11C] (R)-PK11195 in early PD, an *in vivo* marker of microglial activation, reported that reactive microglia are noted in brain regions such as the pons and basal ganglia [63]. These activated microglia then produce a wide range of inflammatory mediators that lead to the continuous death of dopamine neurons. Consistent with this, in PD model mice established by 1-methyl-4-phenyl-1,2,3,6tetrahydropyridine (MPTP), brain microglial activation *via* NLRP3 inflammasomes plays a role in neurotoxicity [64].

Cell-to-cell propagation of α-synuclein aggregates is thought to contribute to the pathogenesis of PD. IL-4-activated microglia effectively reduce the extracellular α-syn and decrease α-syn transfer from neuron to neuron, whereas LPS-treated microglia are less prone to carry out phagocytosis [65]. Pro-inflammatory cytokines can both be neurotoxic and attenuate microglial phagocytosis. LPS, a pro-inflammatory stimulus, attenuates microglial phagocytosis and leads to the increased presence of α-syn within microglia and grafted dopaminergic neurons in a PD mouse model [65]. Croisier *et al*. found that CD68 immunoreactivity is negatively correlated with disease duration, suggesting that elevated phagocytic activity is associated with sustained tissue destruction in PD patients [66]. Therefore, regulating the activation of microglia into a neuroprotective function is a potential therapeutic target.

PD is a chronic disease, making it likely that prevention and treatment will require long-term therapy. Thus, mediating microglia to play a neuroprotection role is a promising field for research.

## Microglia: The Damaging Element in MS

MS is a demyelinating disease of the CNS, including the brain and spinal cord. In MS, demyelination occurs when the immune system inappropriately attacks and destroys myelin, which breaks down communication between neurons, ultimately leading to a variety of sensory, motor, and cognitive problems [67]. MS is a complex and heterogeneous disease with different lesion patterns and mechanisms of tissue injury [68]. MS is also an inflammatory disease of the CNS in which microglia play an important role.

The role of microglia in MS is complex and controversial. Microglia are considered to be damaging elements in MS. Focal lesions of active demyelination and neurodegeneration are characterized by inflammation and microgliosis. A study in an EAE mouse model indicated that microglia are the first cells to take up myelin antigens and subsequently recruit leukocytes to the CNS through MHC class II molecules, thus initiating immune infiltration and the demyelination cascade in the early stage [69]. Microglia also release proinflammatory cytokines such as IL-18 and IL-6 and chemokines to aggravate MS [70]. In MS patients, TMEM119-positive microglia are less abundant in active MS, with restoration associated with disease inactivity [71]. In chronic slowly expanding MS lesions, a ring of activated microglia is also present at the site of lesion expansion [72]. CSF1R signaling (specifically in microglia) is activated in MS and might drive deleterious neuroinflammation, particularly during disease progression [73, 74]. Disruption of remyelination is another pathogenic factor in MS. TREM2 activation on microglia promotes myelin debris clearance and remyelination [75], and *TREM2* genetic deficiency might be a risk factor for MS. Thus, microglia are recognized as key players in MS pathology.

## Perspectives and Reflections on Microglial Replacement Therapy

**Fig. 3:**
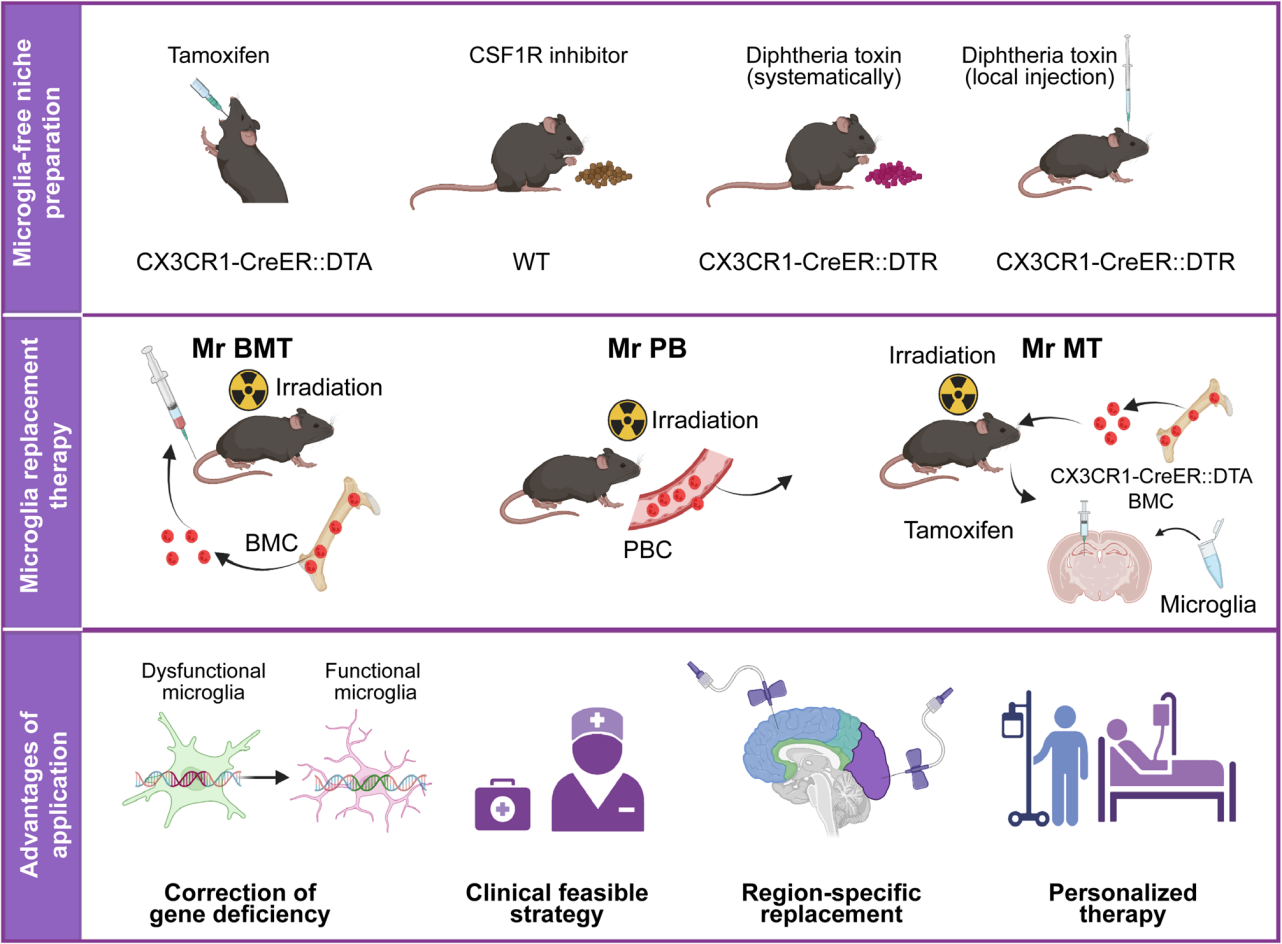
Microglial replacement therapy is a promising strategy for treating neurological disorders. A microglia-free niche is a necessary precondition for microglial replacement. The depletion of microglia is an effective model to create the niche, such as in CX3CR1-CreER::DTA mice. Pharmacological microglial depletion includes the administration of a CSF1R inhibitor or DT in the brain in CX3CR1-CreER::iDTR mice. There are three highly efficient and clinically feasible strategies for microglial replacement in the whole CNS or brain regions of interest: microglial replacement by bone marrow transplantation (Mr BMT), microglial replacement by peripheral blood (Mr PB), and microglial replacement by microglial transplantation (Mr MT). Mr PB clinically boosts donor cell availability. Mr BMT can correct gene deficiency, thus better treating gene-related diseases. Mr MT can achieve region-specific microglial replacement. These three approaches provide options for personalized treatment. DT, diphtheria toxin; DTR, diphtheria toxin receptor; DTA, diphtheria toxin activator. The figure was created with BioRender.com.

As shown above, we demonstrated the role of microglia in the pathology of neurodegenerative disease and the potential therapeutic role of microglia in disease treatment. Some researchers have reported that microglial repopulation can largely resolve the proinflammatory response and promote functional recovery [76]; however, the functional restoration of repopulated microglia depends on exogenous environmental and endogenous genetic changes. Here, we propose that microglia replacement by allogenic cells is an effective method to treat microglia-associated neurodegenerative disorders (Fig. 3).

### The Origin of Microglia

Microglial cells originate from cells produced at ~E7.5 in the yolk sac, at the time of the first wave of hematopoiesis [77, 78]. The second wave starts at E10.5; hematopoietic stem cells (HSCs) are produced in the aorta–gonad–mesonephros region and settle in the fetal liver and other hematopoietic organs of the embryo, where they mature and differentiate into definitive erythrocytes and all myeloid cells, including monocytes. HSCs from the fetal liver finally colonize the bone marrow, the only hematopoietic organ in adults [77]. Due to the specificity of microglial origin, non-autologous cell replacement of microglia may be an effective treatment for microglia-associated neurodegenerative diseases.

### A Microglia-free Niche is a Necessary Precondition for Microglial Re-population

The macrophage niche theory is a concept that first postulates that each macrophage cell occupies its territory [79]. When the niche is available, monocytes can efficiently differentiate into macrophages, and the same process occurs for microglia. The depletion or death of microglia may provide a space that triggers the proliferation of neighboring macrophages to repopulate the space. Microglial niches become temporarily available *via* irradiation-mediated damage or depletion [79]. The irradiation method opens the blood-brain barrier, and this helps a drug to enter the brain through blood circulation and avoids further damage to the CNS. Early approaches depleted microglia by generating mice transgenic for CD11b–thymidine kinase of the herpes simplex virus (HSVTK). In the presence of ganciclovir, HSVTK is activated and induces apoptosis in CD11b^+^ mitotic cells [74]. While CD11b^+^ cells are necessary for normal hematopoiesis and red blood cell production [80], completely depleting them is not a perfect way to deplete microglia. An alternative genetic approach used CX3CR1-CreER::DTA and CX3CR1-CreER::DTR mouse lines to induce diphtheria toxin-dependent microglial cell death [81]. However, DTR models kill only short-lived microglia [81]. Pharmacological microglial depletion can be achieved by targeting the *Csf1r* gene. The CSF1R inhibitors PLX5622, PLX3397, GW5622, and PLX647 are able to cross the brain-blood barrier and induce microglial depletion. Administration of PLX5622 in rodent chow depletes microglia by up to 99% after 2 weeks in adult mice [22]. However, there are still some potential toxic or adverse effects of microglial depletion and replacement. In the report by Rubino *et al*., acute and synchronous microglial depletion in adult mice triggered gray matter gliosis and progressive ataxia-like neurological behavior [82]. Bruttger *et al*. reported that microglial ablation in DTR^MG^ mice leads to a cytokine storm and astrogliosis [81]. Nevertheless, Peng *et al*. reported normal motor performance after diphtheria toxin (DT) treatment in DTR mice [83]. Several papers have reported long-term observations in the PLX depletion model and did not observe motor deficits [21]. The reasons for these different behaviors may involve the dose, concentration, and duration of drug treatment. Therefore, when choosing microglial replacement therapies, we should take into account the potential risks of the method and try to minimize them.

### Current Microglial Replacement Strategies

Microglial replacement therapy has undergone several attempts and failures. Traditional bone marrow transplantation enables the partial replacement of endogenous microglia with donor cells [84]. However, the replacement efficiency is usually <5%–20%, due to the lack of a cell niche [85]. Recent studies established an experimental strategy for transplanting microglia into RAG2^-/-^ IL2Rg^-/-^ recipient mice with clear success. However, the treatment is limited in terms of time and clinical application. In light of these challenges, Xu *et al.* recently developed three effective strategies for microglial replacement either throughout the CNS or focusing only on brain regions of interest [86]: microglial replacement by bone marrow transplantation (Mr BMT) [87], microglial replacement by peripheral blood (Mr PB) [88], and microglial replacement by microglial transplantation (Mr MT) [89]. By choosing an appropriate strategy, the rate of microglial replacement can be effectively boosted. In this protocol, PLX5622 is used to clear microglia, and 9 Gy of whole body irradiation is administered to open the blood-brain barrier. Mr PB is able to induce peripheral blood cells to differentiate into microglia-like cells (MLCs) and can replace 80.74% of resident microglia in the brain. Mr BMT is capable of inducing allografted bone marrow cells (BMCs) to differentiate into microglia-like cells in the entire CNS, replacing 92.66% of resident microglia in the brain. The engrafted microglia after Mr MT exhibit the natural characteristics of naive microglia.

### Similar but not the Same

Microglial replacement combining genetic engineering with cell transplantation represents a cutting-edge approach for the precise treatment of microglia-associated neurodegenerative diseases. Whether transplanted microglia can act as normal microglia is the key issue. Microglial cells in the normal brain are called “true” microglia or *bona fide* microglia, while transplanted microglial cells are often called “microglia-like cells” [90]. This is because of not only their different origins but also their slightly different characteristics. Bone marrow-derived MLCs, when engrafted into the CNS, share ~90% of the transcriptome with host microglia, including the expression of some key microglial genes, such as Tgfb receptor 1 (*Tgfbr1*). In addition, MLCs exhibit their characteristics, as they show reduced gene expression of *Tmem119* and *P2ry12* [91]. Similarly, the MLC population is also maintained by local proliferation [18]. Reports suggest that microglia and MLCs are very similar, and their phenotypes are constantly shaped by the microenvironment in a time- and context-dependent manner. For instance, under pathological conditions, during the acute phase of EAE, microglia shift to a pro-inflammatory macrophage phenotype, while during the chronic phase, macrophages turn down their pro-inflammatory signature to acquire a resting microglia phenotype [92]. However, to our knowledge, the functional differences between microglia and MLCs are not well established; for example, whether they have similar functions in parenchymal surveillance and neuronal circuit refinement remains to be elucidated. We summarize the main benefits and limitations as well as the caveats of each proposed strategy in Table 1. Therefore, these methods offer important rationales for further clinical applications, and each tactic has its own merits and limitations, which provide more choices. Importantly, these methods allow for the genetic modification of replacement microglia and the compensation of functional defects.

**Table 1:** Three methods of microglial replacement and their application.

Strategy	Mr BMT	Mr PB	Mr MT
Source	Bone marrow cells	Peripheral blood	Isolated microglia
Efficiency	92.66% in the brain	80% in the brain	50% in a specified brain region [86]
	99.46% in the retina	74.01% in the retina [86]	
	92.61% in the spinal cord [86]		
Benefits	Replaced microglia are highly dynamic and phagocytic, preserve the immune response.	Replaced microglia are highly dynamic and phagocytic, preserve the immune response, in a fashion similar to microglia [101].	Replaced microglia are highly dynamic and phagocytic, preserve the immune response.
	Ameliorates behavior or cognitive dysfunction and extends survival.	Easily accessible.	Replaced microglia resemble the characteristics of naive microglia.
	Allows for gene editing of donor cells.	CNS-wide replacement.	Allow for local replacement.
	CNS-wide replacement.		
Limitations	Replaced microglia transcriptionally distinct from resident microglia [91, 99].	Replaced microglia transcriptionally distinct from resident microglia [102].	The source is rare.
	Replaced microglia may function differently from microglia [99].	The replacement efficiency of Mr PB is lower than Mr BMT [88].	The procedure for Mr MT is invasive [89].
	Clinically hard to match due to transplant rejection.		Transplanted microglia can migrate to untargeted brain regions.
	Possible immunosuppression and chemotherapy-associated toxicity.		The replacement efficiency is minimal among 3 methods [86].
Application	Disease progression of amyotrophic lateral sclerosis [96]; Alzheimer’s disease [103]; lysosomal and peroxisomal storage diseases [104]	Relatively few studies	Relatively few studies

### Microglial Replacement Therapy in CNS Disease

Microglial replacement therapy has been effectively used to treat other diseases. For instance, transplantation of wild-type bone marrow into *mecp2*-deficient hosts leads to the implantation of bone marrow-derived cells with a microglial phenotype in the brain parenchyma, which arrests disease progression [84]. Another in-depth study indicated that whole bone marrow is heterogeneous and ill-defined, and hematopoietic stem cells play a very important role after Mr BMT transplantation [93]. BM-derived immature monocytic cells can commit to a microglia-like phenotype, and they harbor several features and functions of native microglia [94]. Since resident microglia appear to degenerate in AD, Mr BMT compensates for the deficient functions of senescent resident microglia in AD [95]. ALS is a progressive, fatal neurodegenerative disease. BMT of mSOD1-transgenic mice with BMCs altered the functional properties of microglia and improved the neural cell microenvironment [96].

Moreover, transplantation of genetically modified BM cells can reduce symptoms of CNS diseases. Mr BMT methods are also being continuously improved. Recently, a study developed an approach for rapid and near-complete replacement of microglia in mice with circulation-derived myeloid cells, eliminating the substantial variability that occurs after conventional BMT, which slows neurodegeneration and ameliorates motor dysfunction in prosaposin-mutant mice [93]. However, Mr BMT has its limitations: for example, Mr BMT is a method for whole-brain microglial replacement, not for specific brain regions. Mr MT enables microglial replacement *via* microglial transplantation into the brain area of interest. The method of targeted removal of microglia in specific regions has been studied [97]. Although the application of Mr MT in diseases is relatively rare, microglial recolonization in a brain region of interest will certainly have better application prospects. We speculate that therapeutic genes can be transduced into the stem or progenitor cells with lentiviral vectors, leading to stable integration of genes such as *MeCP2* in Rett syndrome or *TREM2* in AD mice. It is conceivable that by genetically modifying the extracted normal microglia, we can treat more intractable genetic defects and avoid immune rejection at the same time.

## Targeting Microglial Therapeutics: Challenges and Opportunities

In this review, we summarized the critical pathological role of microglia in several neurodegenerative diseases, mostly focused on AD. We envision microglial replacement as a potential medical intervention for many neurological diseases such as PD and MS, but further research is needed.

What factors affect the replacement of microglia? Xu *et al*. reported that Mr BMT cells are mainly derived from CCR2-positive BMCs [86]. CCR2-positive cells are migratory cells that respond to neural insults [98]. Therefore, the function of CCR2 may be related to the mechanism of microglial replacement in the brain. In addition, a study has indicated that engrafted BM-derived myeloid cells display significantly increased levels of CD68, a lysosomal marker associated with a heightened activation or phagocytic state [99]; microglial engraftment affects astrocyte activation and neuronal communication [99]. Thus, molecules that affect microglial activation or interactions with other cells, such as CX3CR1/CX3CL1 and CD200/CD200R [100], might also affect microglial replacement. The removal rate of resident microglia directly affects the replacement efficiency of external them. Our recent research found that microglial debris is primarily removed by astrocytes in the brain *via* the opsonization of C4b [105]. Microglia engraftment likely affects astrocyte reactivation. Additional research into the mechanisms of microglial replacement would be beneficial for developing clinical therapies.
