# Correction: Novel Microglia-based Therapeutic Approaches to Neurodegenerative Disorders

**DOI:** 10.1007/s12264-023-01026-9

**Published:** 2023-02-13

**Authors:** Lijuan Zhang, Yafei Wang, Taohui Liu, Ying Mao, Bo Peng

**Affiliations:** 1 Department of Neurosurgery, Huashan Hospital, Institute for Translational Brain Research, State Key Laboratory of Medical Neurobiology, MOE Frontiers Center for Brain Science, Fudan University, Shanghai, 200040 China; 2 Co-innovation Center of Neuroregeneration, Nantong University, Nantong, 226001 China

**Correction: Neurosci. Bull.** https://doi.org/10.1007/s12264-022-01013-6

The author contribution was missing from this article and should have read ‘Lijuan Zhang, Yafei Wang, Taohui Liu contributed equally to this review.’

The original article has been corrected.

